# Air-IFF-PINN: an Air-Pollution Inverse-Forward Fourier Physics-Informed Neural Network framework for reconstructing and imputing satellite-derived pollutant fields

**DOI:** 10.1016/j.mex.2026.104176

**Published:** 2026-09-27

**Authors:** Helena Margaretha, Nicholas Andrew Tanubrata, Kie Van Ivanky Saputra, Ferry Vicenttius Ferdinand, Ardhasena Sopaheluwakan

**Affiliations:** a Department of Mathematics, Faculty of Science and Technology, Universitas Pelita Harapan, Tangerang, 15811, Indonesia; b Agency for Meteorology, Climatology and Geophysics (BMKG), Jakarta, 10610, Indonesia

**Keywords:** PINNs, Inverse problem, Forward problem, Sentinel-5P, Multi-mode Fourier feature layer

## Abstract

Satellite-derived air pollution datasets often contain missing values caused by retrieval limitations, cloud cover, and uneven spatial-temporal coverage. This article presents Air-IFF-PINN, an Air-Pollution Inverse-Forward Fourier Physics-Informed Neural Network framework for reconstructing and imputing incomplete pollutant fields when key physical parameters are unknown. The method combines an advection-diffusion formulation, a multi-mode Fourier feature PINN architecture, and a staged inverse-forward training strategy. In the inverse stage, the effective diffusion parameter is estimated from incomplete observations. In the forward stage, the estimated parameter is used to reconstruct missing satellite-derived pollutant values within the observed spatial-temporal domain. The workflow is demonstrated using Sentinel-5P/TROPOMI Level-3 carbon monoxide data and ERA5-Land wind fields over the Jakarta Metropolitan Area during a major fire event. The method provides a reproducible framework for handling incomplete satellite observations, estimating unknown PDE parameters, and generating physically coherent pollutant reconstructions within the observed spatial-temporal domain.

## Abstract (author highlights)

• Integrates inverse parameter estimation and forward pollutant reconstruction within one PINN workflow.
• Uses multi-mode Fourier features to capture pollutant patterns at different spatial and temporal scales.
• Provides a reproducible workflow for imputing and reconstructing missing satellite-derived air pollution data under incomplete observations.

## Graphical abstract

**Figure:**
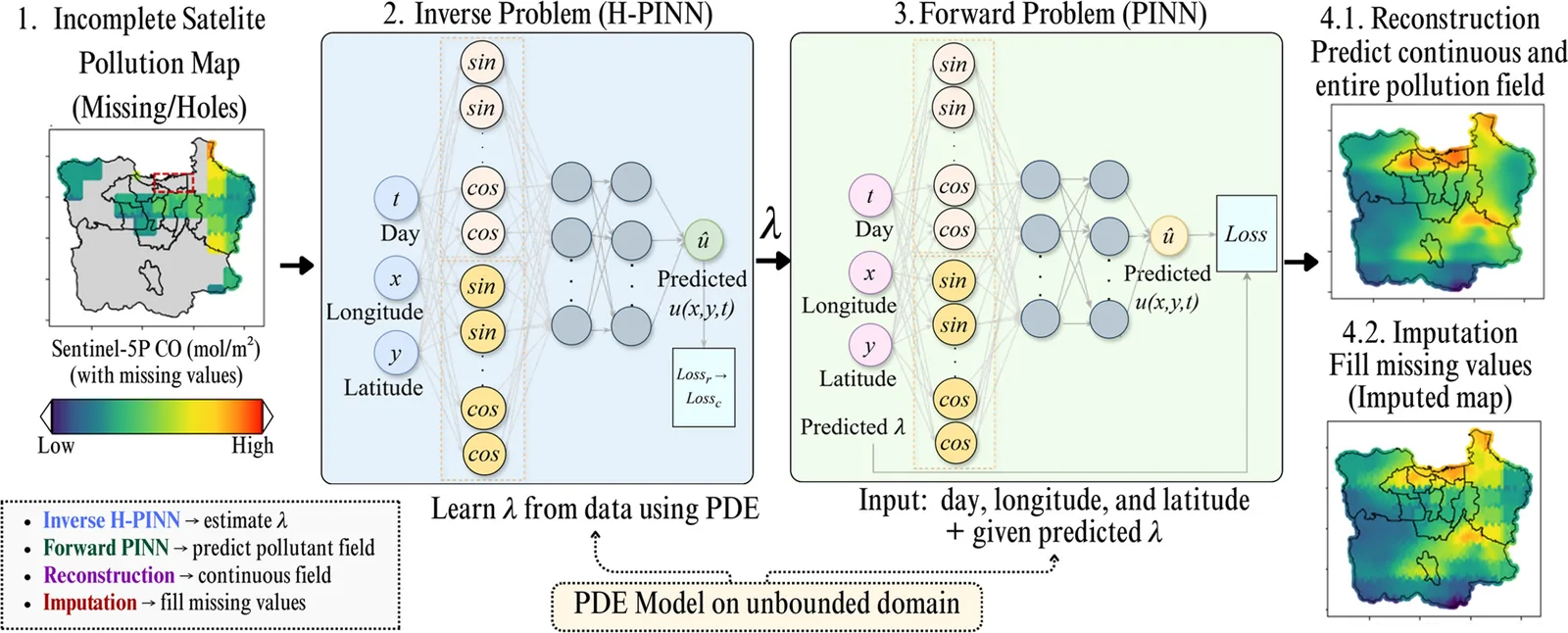

## Background

Accurate reconstruction of spatiotemporal air pollutant fields remains challenging when observations are sparse, incomplete, and unevenly distributed. Ground-based monitoring stations provide reliable temporal measurements but limited spatial coverage [1], while satellite observations offer broader spatial information but are often affected by missing data due to cloud cover and retrieval constraints. As a result, pollutant datasets commonly contain substantial gaps.

A central difficulty is that missing data reconstruction and parameter estimation must often be performed simultaneously. In practical applications, key parameters in the governing physical model, such as diffusion or transport coefficients, are typically unknown and cannot be reliably specified in advance [2]. Consequently, reconstructing missing pollutant values is coupled with the inverse problem of estimating unknown physical parameters. This makes the problem ill-posed, since both the pollutant field and model parameters must be inferred from incomplete observations.

Physics-Informed Neural Networks (PINNs) [3] provide a natural framework for this setting by embedding governing partial differential equations (PDEs) into the learning process. In principle, PINNs enable simultaneous field reconstruction and parameter estimation. However, their direct application to environmental datasets remains difficult due to unstable inverse parameter estimation, sensitivity to initialization, and limited ability to represent high-frequency spatial variations. Existing studies also often focus on either forward simulation or inverse estimation separately, without explicitly addressing missing data reconstruction under unknown parameter conditions.

Several developments address parts of these challenges. Fourier feature embeddings improve the representation of high-frequency and multiscale PDE solutions [[4], [5], [6]], while structured training strategies, such as Hadamard-based PINN (H-PINN) formulations [7], enhance convergence in inverse problems with distant initial parameter guesses. However, these approaches are typically applied separately.

In addition, incorporating detailed chemical reactions into the governing PDE introduces additional parameters and state variables, increasing the dimensionality of the inverse problem. Under sparse and incomplete observations, this may lead to instability and poor parameter identifiability. Therefore, a reduced-order PDE formulation is needed to support stable reconstruction while keeping the problem tractable.

This study proposes a structured Air-IFF-PINN workflow that combines PINN-based modeling, multi-mode Fourier feature representations, and a staged inverse-forward training strategy to reconstruct missing pollutant values and estimate unknown PDE parameters. The workflow integrates data preprocessing, parameter estimation, statistical validation, and forward prediction within a single framework. By adopting an advection-diffusion formulation, the method maintains tractability while capturing dominant spatiotemporal pollutant behavior.

Another practical advantage is that the governing advection-diffusion PDE can be imposed through residual loss at sampled spatiotemporal points, without explicit mesh-based discretization of the full atmospheric domain. Unlike conventional numerical treatments of open or unbounded domains, which often require artificial or transparent boundary conditions, the present PINN workflow encourages physical consistency within a finite observational window using data loss and PDE residual loss.

While the methodology is designed to be applicable across pollutants, spatial resolutions, and event types, this article focuses on a representative case study to demonstrate implementation and reproducibility.

### Method details

The overall experimental workflow in this study is illustrated in Fig. 1. It consists of data extraction, preprocessing, and splitting into training and testing data, followed by model setup, which includes the PINN architecture, loss function, optimizer, and learning rate scheduler. This process is followed by inverse problem training to estimate the effective diffusion parameter, forward problem training to predict pollutant concentrations, and finally, reconstruction and imputation.

**Fig. 1:**
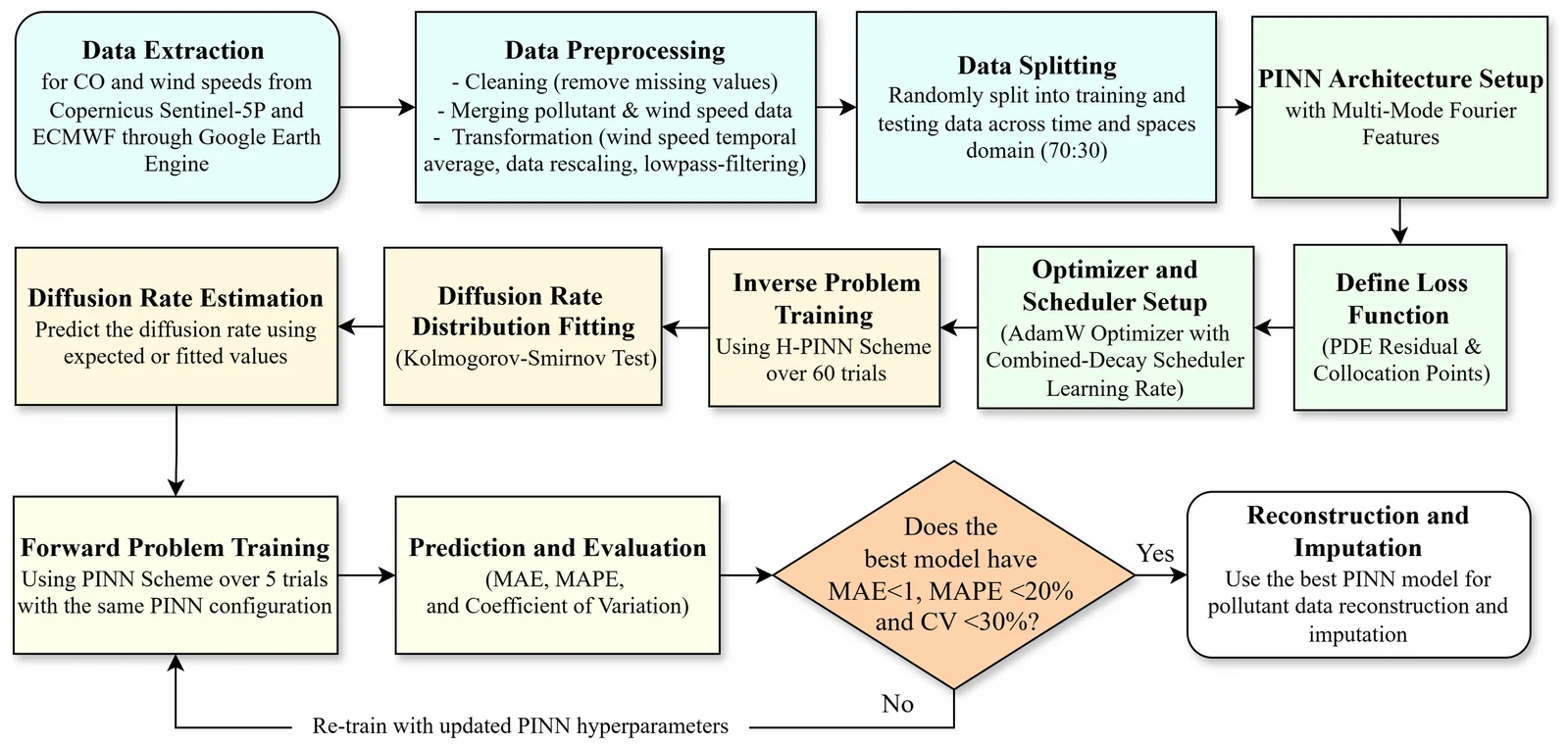
Workflow of the PINN framework for pollutant data reconstruction and imputation.

### Dataset sources

The pollutant data were obtained from Sentinel-5P/TROPOMI, which provides satellite-based observations of atmospheric trace gases in units of mol/m² [8,9]. In this study, the COPERNICUS_S5P_OFFL_L3_CO product available through Google Earth Engine was used to obtain Level-3 gridded CO concentrations [10]. The Level-3 product was selected because it provides spatially gridded data derived from the original Level-2 observations, making it suitable for extraction over a fixed analysis grid.

To account for atmospheric transport, eastward (*wₓ*) and northward (*wᵧ*) wind speeds at 10 m height were obtained from the ECMWF_ERA5_LAND_HOURLY dataset in Google Earth Engine [11,12]. Wind values were sampled at the pollutant grid coordinates to align the meteorological and pollutant datasets. This procedure assigns ERA5-Land wind values to the corresponding pollutant grid cells without additional spatial downscaling.

### Data extraction and preprocessing

To demonstrate the proposed method, we present a representative case: the reconstruction of CO concentrations over a fine spatial grid in the Jakarta Metropolitan Area during a major fire event. The COPERNICUS_S5P_OFFL_L3_CO data products were employed, providing Sentinel-5P Level-3 gridded concentrations of CO derived from Google Earth Engine [10].

During the data extraction process, the Google Earth Engine Level-3 product was sampled onto the analysis grid. Pollutant values falling within each grid cell were averaged, and the centroid of the grid cell was used as the representative latitude and longitude. This step was performed to harmonize the spatial representation of the satellite-derived observations and to obtain a consistent gridded dataset for PINN training. It should not be interpreted as physical downscaling beyond the information content of the original satellite product.

The raw data contain substantial missing values. This issue is partly related to satellite retrieval limitations, since spaceborne sensors may not retrieve valid observations under cloud-covered conditions and other unfavorable retrieval settings [13].

Although pollutant concentration data are available for certain days and locations, the corresponding wind speed data are often incomplete. Therefore, the data required several preprocessing steps to ensure data quality and consistency before model training. These steps included data cleaning and merging between pollutant and wind speed datasets; averaging wind speed over time for each spatial coordinate; rescaling the variables; and noise reduction through low-pass filtering.

### Data cleaning and merging

The data cleaning process began with the removal of missing values from both pollutant concentrations and wind speed records. Negative pollutant concentration values were treated as invalid. Subsequently, the wind speed and pollutant datasets were merged into a single data frame using an inner join on matching time and location, ensuring that each observation contained both variables on the same day and at the same location. The remaining observations (with either missing or invalid pollutant data or wind speed data) were excluded from model training and later incorporated into the reconstruction process using the trained PINN models.

### Wind speed averaging

The wind velocity field is approximated as time-independent by averaging the wind speed over the observation period at each spatial location. This approximation is introduced to maintain consistency with the governing advection-diffusion formulation and to reduce the dimensionality of the inverse problem.

Incorporating fully time-dependent wind fields would increase the temporal variability of the prescribed input field and make the optimization process more sensitive, particularly under sparse and incomplete observations. This may lead to additional challenges in parameter identifiability and training stability within the PINN framework [2,14]. Therefore, the time-averaged velocity field is used, as it still preserves the main transport direction, while allowing the PINN to reconstruct the pollutant field and estimate the related parameters.

### Data rescaling

Data rescaling was applied to both the spatial coordinates and pollutant concentrations. For latitude and longitude, rescaling was performed by multiplying each coordinate by 100. This choice was motivated by the fact that one degree corresponds to approximately 111.1 km for both latitude and longitude at the equator0F.^2^

Small decimal changes in coordinates thus represent substantial spatial variation, and scaling in this manner helps the PINN better capture their significance. Furthermore, for pollutant concentrations, the original median magnitudes of CO concentrations in the Jakarta Metropolitan Area were on the order of $10^{-2}$. These scales cause the model to struggle in minimizing the mean squared error (MSE, as detailed in the next section) of the data.

Since absolute errors below 1, such as those in the range of $10^{-1}$ to $10^{-5}$, become even smaller when squared, the loss function assigns relatively small penalties to these deviations and thus struggles to detect their significance during training. For instance, if the actual pollutant concentration is 1 ×$10^{-4}\;$and the PINN prediction is 1 ×$10^{-5}$, the absolute error is 9 ×$10^{-5}$. When squared in the MSE, it will become 8.1 ×$10^{-9}$.

The model underestimates the significance of such deviations. To address this, the concentrations were rescaled so that the errors become numerically larger and therefore more influential during training. Accordingly, CO concentrations were divided by a scaling factor of $10^{-2}\;$during model training.

### Fourier transformation in low-pass filtering

To remove high-frequency fluctuations while preserving long-term variations in the pollutant time series, a low-pass filter based on the Fourier transform was applied. This filtering step reduces short-term noise or volatility in pollutant concentrations over time at each fixed spatial coordinate.

Let u(t) denote the pollutant concentration time series at a fixed spatial coordinate, and let F(s) be its Fourier transform. A cutoff frequency $f_{c}$ was applied through the filter function

$$H\left(s\right)=\left\{ \begin{array}{cc} 1, & \left|s\right|<f_{c}\\ 0, & \left|s\right|\geq f_{c}. \end{array}\right.$$

The filtered Fourier representation was then defined as W(s)=F(s)H(s) and the filtered pollutant series $u^{filtered}\left(t\right)$ was obtained by applying the inverse Fourier transform to W(s). The cutoff value was selected from the training data by evaluating the residuals between the original and filtered pollutant series and choosing the value that produced approximately Gaussian residuals according to the Kolmogorov-Smirnov test. The selected cutoff was then fixed and applied consistently to all spatial coordinates in the CO dataset. For the CO case study, the selected value was $f_{c}=0.1$, and the Kolmogorov-Smirnov test did not reject the normality of the residuals at the significance level α=1%.

### Data splitting

Furthermore, the preprocessed data are split randomly into 70% training data to optimize the PINN parameters and 30% testing data to assess the model performance in data validation. Collocation points are sampled randomly from the available spatio-temporal domain based on the dataset size and remain fixed during training. The random split evaluates interpolation performance within the observed spatiotemporal domain.

### PINN architecture setup

The PINN architecture was designed to approximate pollutant concentration as a function of time, longitude, and latitude. The governing advection-diffusion equation was incorporated into the loss function through automatic differentiation [15]. In both the inverse and forward stages, the neural network uses multi-mode Fourier feature embeddings to represent multiscale spatial-temporal pollutant patterns, as illustrated in Fig. 1. This configuration is motivated by the multiscale nature of pollutant fields. By introducing multiple frequency modes into the input representation, the architecture is designed to represent spatial-temporal variations across different frequency scales, following the motivation of Fourier-feature PINNs for multiscale PDE problems [[4], [5], [6]].

### Multi-mode fourier feature layer

One of the challenges in the standard PINN architecture is spectral bias, which refers to the tendency of the model to struggle in learning high-frequency patterns or complex functions. Therefore, PINNs with Fourier features are designed to improve the representation of high-frequency functions or multi-scale problems [5]. The distinction between this architecture and a standard PINN lies in the initial layer of the neural network. A PINN with Fourier features adds a layer containing trigonometric functions, namely sine and cosine vectors, between the input layer and the first hidden layer. The Fourier feature introduced by [4] was defined as:

(1) $$\bar{X^{\left(2\right)}}=\left[\sin\left(W_{\sigma }^{\;\left(1\right)}\bar{X^{\left(1\right)}}\right)\cos\left(W_{\sigma }^{\;\left(1\right)}\bar{X^{\left(1\right)}}\right)\right],$$

which corresponds to a single-mode representation, where the frequency scaling is controlled by a set of parameters $W_{\sigma }^{\;(1)}\in R^{m\times n}$ sampled from a single Gaussian distribution $N(0,\sigma^{2})$ [4].

While this formulation improves the ability of the network to capture the periodic component, all features are projected onto a single frequency distribution scale, $\sigma^{2}$. In the context of inverse problems, where the model must simultaneously reconstruct missing data and estimate unknown PDE parameters for pollutant fields containing variations at multiple spatial-temporal scales, we therefore extend this formulation by using multiple frequency scales. In the multi-mode Fourier features, $W_{\sigma }^{\;(1)}$is extended with multiple hyperparameters from ${\sigma_{1}}^{2}$ to ${\sigma_{n}}^{2}$, leading to the following modified formulation:

(2) $$\bar{X^{\left(2\right)}}=\left[\sin\left(W_{\sigma_{1}}^{\;\left(1\right)}\bar{X^{\left(1\right)}}\right)\cos\left(W_{\sigma_{1}}^{\;\left(1\right)}\bar{X^{\left(1\right)}}\right)\sin\left(W_{\sigma_{2}}^{\;\left(1\right)}\bar{X^{\left(1\right)}}\right)\cos\left(W_{\sigma_{2}}^{\;\left(1\right)}\bar{X^{\left(1\right)}}\right)\;\cdots \;\sin\left(W_{\sigma_{n}}^{\;\left(1\right)}\bar{X^{\left(1\right)}}\right)\cos\left(W_{\sigma_{n}}^{\;\left(1\right)}\bar{X^{\left(1\right)}}\right)\right].$$

Here, $W_{\sigma_{1}}^{\;(1)},W_{\sigma_{2}}^{\;(1)},\ldots ,W_{\sigma_{n}}^{\;(1)}$ denote trainable Fourier feature weight matrices. Each matrix is initialized from a Gaussian distribution with its corresponding variance, $N\left(0,\sigma_{1}^{2}\right),N\left(0,\sigma_{2}^{2}\right),\ldots ,N\left(0,\sigma_{n}^{2}\right)$*,* respectively, and then updated during training together with the neural network parameters*.* The multi-mode architecture is presented in Fig. 2. The hyperparameter options for this architecture that are applied in our numerical experiments are summarized in Table 1.

**Fig. 2:**
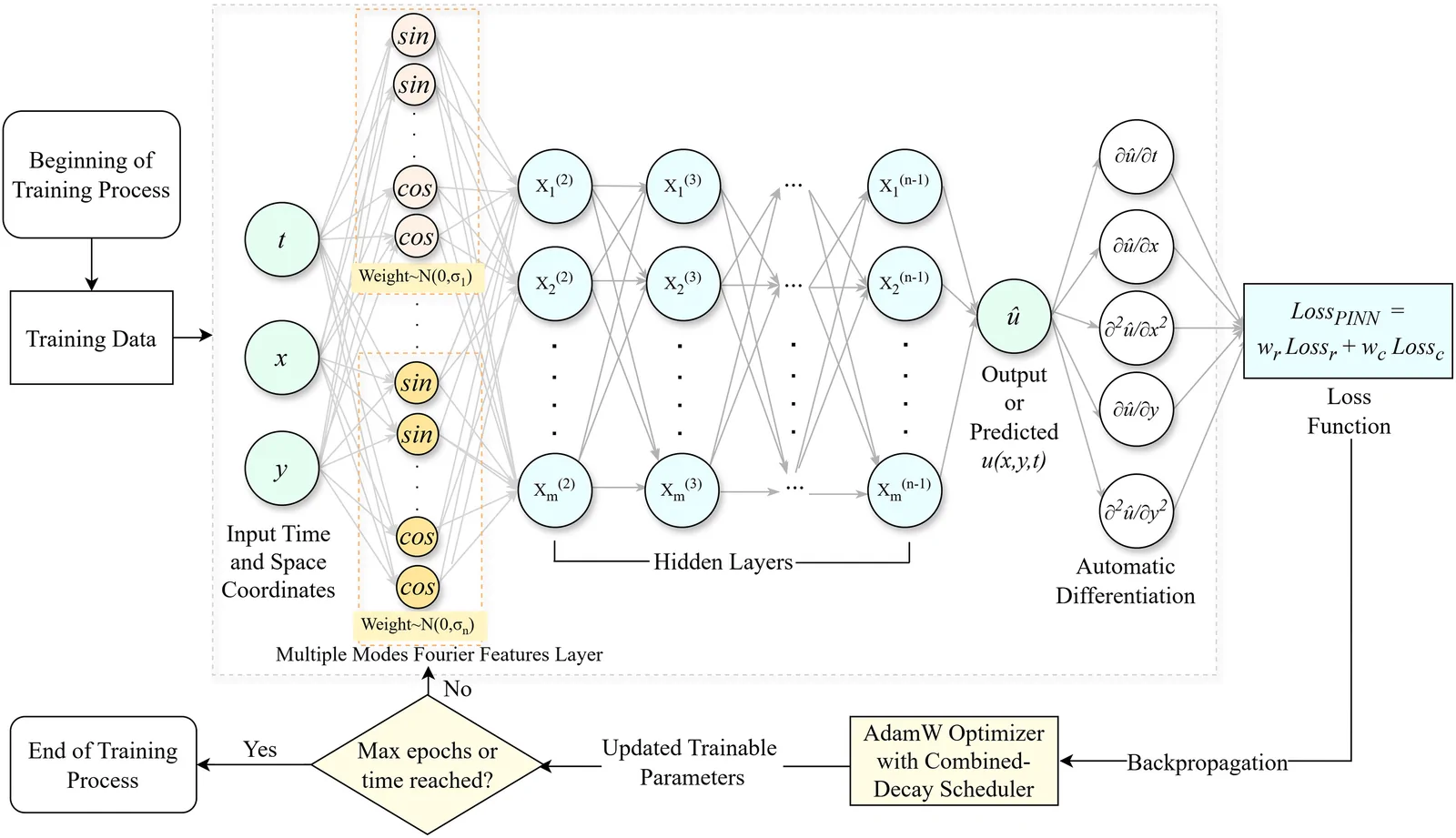
Architecture of PINNs with a multi-mode Fourier feature layer.

**Table 1:** Hyperparameters of the PINN architectures used in this study.

Hyperparameter	Multi-mode Fourier Feature Layer Fourier feature output dimension: 256 Fourier-to-hidden mapping: 256 Fourier features to 100 hidden neurons
Network depth	5 layers
Network width	100 neurons/layer
Additional layer(s) depth	Multi-mode Fourier feature layer with $\sigma_{1}=1.0$ and $\sigma_{2}=2.0$
Additional layer(s) width (in total)	256 neurons
Activation function (hidden layers)	tanh, Swish
Activation function (output layer)	linear, Softplus

The multi-mode Fourier feature layer contains 3 × 256=768 trainable weight parameters, corresponding to the three input variables and 256 Fourier-feature nodes. The resulting 256-dimensional feature representation is then mapped to the first hidden layer, which contains 256*n* weights and *n* biases. Each subsequent hidden layer contains *n*^2^ wt and *n* biases, while the output layer contains *n* weights and one bias. Therefore, the total number of trainable parameters in the forward PINN is

$$768+\left(256n+n\right)+4\left(n^{2}+n\right)+\left(n+1\right)=4n^{2}+262n+769.$$

For the inverse PINN, the effective diffusion parameter λ is also trainable, resulting in $4n^{2}+262n+770$. With n = 100, the forward and inverse PINNs contain 66,969 and 66,970 trainable parameters, respectively.

### Define loss functions and mathematical models

The loss function combines the PDE and the collocation point loss, where the PDE loss is derived from the advection-diffusion PDE. This PDE model accounts for both the effective diffusion parameter and wind velocity over a two-dimensional spatial domain. The wind velocity in this study is assumed to be time-independent and is represented by its average over time. By adjusting several notations, the PDE can be expressed as follows [16]:

(3) $$F\left[u\left(x,y,t\right);\lambda \right]\;=\;\frac{\partial u}{\partial t}+w_{x}\left(x,y\right)\frac{\partial u}{\partial x}+w_{y}\left(x,y\right)\frac{\partial u}{\partial y}-\lambda \left(\frac{\partial^{2}u}{\partial x^{2}}+\frac{\partial^{2}u}{\partial y^{2}}\right)=0$$

Here, u(t,x,y) is the pollutant concentration at day t and spatial position (x,y), x denotes the horizontal coordinate (longitude) and y the vertical coordinate (latitude), $w_{x}$ and $w_{y}$ are the wind velocities in the x and y directions, respectively, and λ is the effective diffusion parameter. The x-axis is positive from west to east, while the y-axis is positive from south to north. In the present reduced-order formulation, an explicit pollutant source term is not included; the implications of this assumption for the fire-event case are discussed in the reconstruction section.

In this implementation, u(t,x,y) represents the Sentinel-5P CO column density rather than a local near-surface concentration. The column density is treated as a two-dimensional scalar field whose horizontal evolution is approximated by the advection-diffusion equation. Accordingly, *λ* is interpreted as an effective diffusion parameter, while the ERA5-Land 10 m wind field serves as a proxy for horizontal transport. This reduced-order formulation does not resolve vertical transport within the atmospheric column.

Since the model output was rescaled, the pollutant concentration is transformed as

(4) $$u_{\text{scaled}}\left(t,x,y\right)=\frac{u\left(t,x,y\right)}{scale}$$

Substituting into the advection-diffusion equation yields

(5) $$F\left[u\left(x,y,t\right);\lambda \right]\;=\;scale\cdot \left(\frac{\partial u_{\text{scaled}}}{\partial t}+w_{x}\left(x,y\right)\frac{\partial u_{\text{scaled}}}{\partial x}+w_{y}\left(x,y\right)\frac{\partial u_{\text{scaled}}}{\partial y}-\lambda \left(\frac{\partial^{2}u_{\text{scaled}}}{\partial x^{2}}+\frac{\partial^{2}u_{\text{scaled}}}{\partial y^{2}}\right)\right)=0$$

In this study, the loss function is divided into two scenarios: the inverse problem and the forward problem. For the forward problem, the loss function is modified from [17], and is defined as:

(6) $$L\left(\theta \right)=w_{r}L_{r}\left(\theta \right)+w_{c}L_{c}\left(\theta \right)$$

Here, $L_{r}\left(\theta \right)$ represents the residual loss, which quantifies the model's deviation from the governing PDE at interior collocation points, and $L_{c}\left(\theta \right)$ corresponds to the concentration data loss at observed points. In the equation, $w_{r}$ and $w_{c}$ are weighting factors that balance the contributions of these two terms. In this study, $w_{r}$ was set to 10.0, and $w_{c}$ was initialized to 1.0.

The components of $L_{r}\left(\theta \right)$ and $L_{c}\left(\theta \right)$ are defined as:

(7) $$L_{r}\left(\theta \right)=\frac{1}{N}\sum_{k=1}^{N}{\left(F\left[\hat{u}\left(x_{k}^{*},y_{k}^{*},t_{k}^{*};\theta \right);\lambda \right]\right)}^{2},\;L_{c}\left(\theta \right)=\frac{1}{N}\sum_{k=1}^{N}{\left(u\left(x_{k}^{*},y_{k}^{*},t_{k}^{*}\right)-\hat{u}\left(x_{k}^{*},y_{k}^{*},t_{k}^{*}\right)\right)}^{2}$$

In these expressions, θ denotes the trainable parameters of the neural network; u(x,y,t;θ) represents the pollutant concentrations at known spatial and temporal points, while $\hat{u}\left(x,y,t;\theta \right)$ is the predicted concentrations. F[·;λ] stands for the differential operator with coefficients λ. The coordinates $\left(x_{k}^{*},y_{k}^{*},t_{k}^{*}\right)$ correspond to the spatial and temporal locations of the collocation points, and N denotes the number of sampling points used for the collocation points.

In the inverse problem, $L_{r}\left(\theta \right)$ and $L_{c}\left(\theta \right)$ were trained separately: first by minimizing $L_{c}\left(\theta \right)$, and then by minimizing $L_{r}\left(\theta \right)$. Additionally, to prevent unrealistically low estimates of λ, a squared hinge penalty is applied whenever the estimated effective diffusion parameter $\left(\hat{\lambda }\right)$ falls below $1\times 10^{-3}$. Consequently, this study employs the modified PDE loss $\left({L^{\prime }}_{r}\left(\theta \right)\right)$ as:

(8) $${L^{\prime }}_{r}\left(\theta \right)=L_{r}\left(\theta \right)+\alpha \cdot \max{\left(0,\text{threshold}-\hat{\lambda }\right)}^{2}$$

where the threshold is the minimum acceptable value of λ, set to $1\times 10^{-3}$, and α is the penalty weight, set to $1\times 10^{-2}$.

### Optimizer and scheduler setup

Training is performed using the AdamW optimizer with a combined exponential-piecewise decay learning rate scheduler [18]. In this scheme, the update parameter is modified as [19]:

(9) $$\Theta_{k}\leftarrow \Theta_{k-1}-\alpha \cdot \left(\frac{\hat{m_{k}}}{\sqrt{\hat{v_{k}}}+\varepsilon }+\lambda_{penalty}\cdot \Theta_{k-1}\right),\forall \epsilon >0.$$

The $\lambda_{penalty}$ denotes the weight decay coefficient, which controls the magnitude of the penalty on the model parameters. In this study, $\lambda_{penalty}$ is initialized with $1\times 10^{-4}$ for the inverse problem and $1\times 10^{-3}$ for the forward problem.

This study implements a learning rate scheduler that combines exponential decay with piecewise constant decay [7]. The scheme begins with an exponential decay and transitions to a piecewise constant decay, where each stage uses a value that is half of the previous learning rate. The initial learning rate is set to $1\;\times 10^{-2}$, with decay steps of 1000 and a decay rate of 0.9. Furthermore, the learning rate is halved at steps 7000; 10,000; 11,000; 13,000; 15,000; and 20,000.

### Inverse problem for diffusion rate

Model training begins with the inverse problem, in which the effective diffusion parameter is estimated for each region before predicting pollutant concentrations, serving as initialization for the forward problem in the PINNs. Since the ground-truth λ value is unknown in real-world scenarios, this study applies the H-PINN scheme, as illustrated in Fig. 3. An initial guess of λ=0.5 is provided to guide the convergence during training. The procedure starts by fitting the neural network to the data to optimize its parameters. The derivatives of the predicted values, obtained via automatic differentiation, are then used to estimate λ [7]. In practice, the data loss $L_{c}\left(\theta \right)$ in Eq. 7 is minimized first, followed by the modified PDE residual loss $L_{r}^{\prime }\left(\theta \right)$ in Eq. 8. Approximately 80% of the training time is allocated to minimizing the data loss, with the remaining 20% devoted to the PDE loss.

**Fig. 3:**
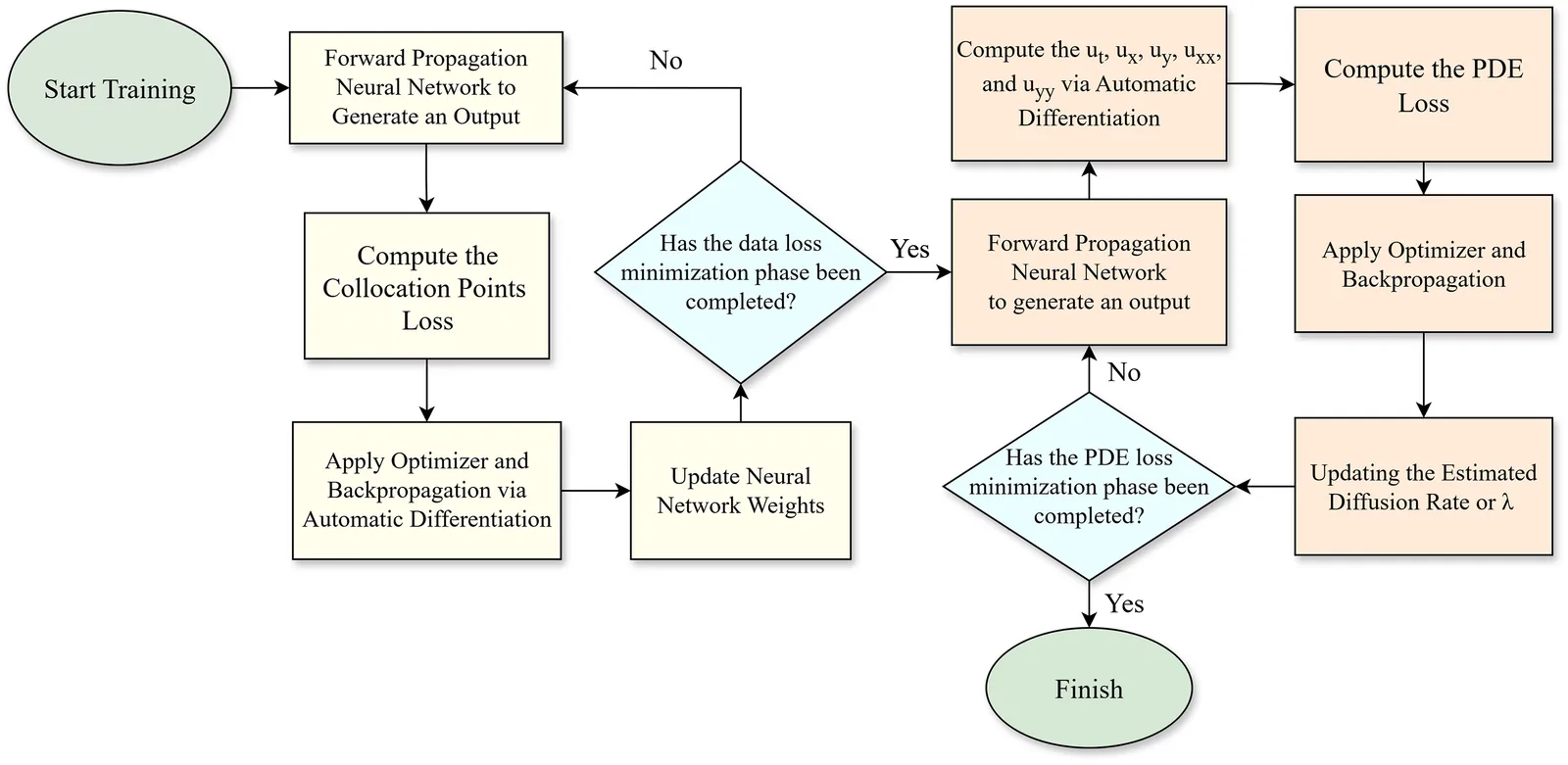
Workflow of the H-PINN scheme for an inverse problem.

Due to the stochastic nature of the PINN resulting from random weight initialization, this scheme was implemented over 60 independent trials. This approach allows the central tendency and variability of the estimated parameter to be assessed. The 60 estimated values were fitted to seven candidate probability distributions using SciPy: Normal, Beta, Exponential, Burr Type III, Fisk, Inverse Gaussian, and Generalized Gamma. The fitted distributions were evaluated using the Kolmogorov-Smirnov test, and the expected value of the selected distribution was used as the effective diffusion parameter in the subsequent forward problem, where the PINNs minimize Eq. 6 to predict pollutant concentrations.

### Forward problem for air pollution prediction

After the inverse stage, the estimated diffusion parameter $\hat{\lambda }$ was substituted into the PDE residual term of the forward PINN. The forward model was then trained to predict pollutant concentrations by minimizing the total loss in Eq. 6. Each forward training attempt was run for 20 min using time-based epochs.

### Prediction on testing data and evaluation

After training the forward problem, the PINN predictions on the testing data are evaluated against the ground truth using MAE, MAPE, and the Coefficient of Variation (CV) on the rescaled data. In this study, the Mean Absolute Error (MAE) will be used to quantify the absolute difference between the PINN predictions and the actual air pollutant concentrations. The MAE is defined as

(10) $$\text{MAE}=\frac{1}{N}\sum_{i=1}^{N}\left|u\left(x_{i},y_{i},t_{i}\right)-\hat{u}\left(x_{i},y_{i},t_{i}\right)\right|,$$

where u(x,y,t) represents the actual pollution data, $\hat{u}\left(x,y,t\right)$ denotes the PINN-predicted-values, and N is the number of testing observations. To compute the relative error of the PINN models, Mean Absolute Percentage Error (MAPE) is used and defined as

(11) $$\text{MAPE}=\frac{1}{N}\sum_{i=1}^{N}\left|\frac{u\left(x_{i},y_{i},t_{i}\right)-\hat{u}\left(x_{i},y_{i},t_{i}\right)}{u\left(x_{i},y_{i},t_{i}\right)}\right|\times \;100\%.$$

To assess the stability of model performance across repeated runs, we report the mean and standard deviation of each error metric. CV is also reported as a descriptive relative variability measure:

(12) $$\text{CV}=\frac{SD}{Mean}\times \;100\%$$

where SD denotes the standard deviation and Mean is the average of the estimated values.

As practical acceptance criteria for this case study, we used: MAE < 1 upon rescaling the pollution data, MAPE < 20%, which are considered indicators of good predictive accuracy in the environmental science context [20]. To assess the stability of model performance across repeated runs, we report the mean and standard deviation of each error metric. CV is additionally reported as a descriptive measure of relative variability. A CV threshold of 30% has been used as a precision criterion in field evaluations of atmospheric gas measurement sensors [21]. However, this criterion was developed for measurement-device reproducibility rather than numerical experiments. Therefore, in the present study, CV < 30% is adopted only as a heuristic indicator of acceptable variability across repeated PINN runs.

In the proposed method, if any of the MAE, MAPE, or CV criteria are not met, the PINN hyperparameters are adjusted to improve performance. Once validated, the model can then be used for the reconstruction and imputation of the preprocessed pollution data by providing the specified day, latitude, and longitude.

### Method validation

Although the primary objective of this article is to present the reconstruction workflow rather than an extensive benchmarking study, several validation procedures were conducted to evaluate the stability, consistency, and practical applicability of the proposed methodology. The validation focuses on two main aspects: (i) the stability of inverse parameter estimation and (ii) the consistency of pollutant reconstruction under incomplete observations.

The main validation case involves CO reconstruction over the Jakarta Metropolitan Area during a major fire event. During method development, the workflow was also explored under multiple configurations, including different pollutants (CO and NO₂), spatial domains (Western Java centroids, Jakarta Metropolitan Area, and Bangkok City), and event scenarios, such as traffic-related and fire events. Implementation scripts, additional exploratory experiments, and reproducibility materials are provided in the accompanying GitHub repository, with the link given in the supplementary material section.

For the inverse problem, the training process was repeated over 60 independent trials to evaluate the distribution and stability of the estimated effective diffusion parameter in the advection-diffusion PDE. Each trial for the Jakarta Metropolitan Area required approximately 300 s of computation time. For the forward problem, each model configuration was run for five attempts, with each attempt taking 1200 s. In the Jakarta Metropolitan Area, the training scheme used a batch size of 4096 to handle the large dataset while balancing the optimization of data-fitting and physics-based constraints.

### Data preprocessing outcomes

Before presenting the modeling performance, we summarize the data characteristics after preprocessing. Table 2 reports the number of samples, mean, and standard deviation of each variable at each preprocessing step. Wind variables were spatially matched to the pollutant grid before training.

**Table 2:** Data counts and summary statistics for CO in the Jakarta Metropolitan Area at each preprocessing step, including the wind speed associated with each grid.

Step	Variable	Count	Mean	Standard Deviation
Raw observations	CO $w_{x}$ $w_{y}$	2223,580 2223,580 2223,580	3*.*49 × 10^–2^ 1*.*55 × 10^–1^–2*.*49 × 10^–2^	7*.*15 × 10^–3^ 1.0319 5 × 10^–1^
After cleaning	CO $w_{x}$ $w_{y}$	1355,558 2088,530 2088,530	3*.*49 × 10^–2^ 1*.*55 × 10^–1^–2*.*49 × 10^–2^	7*.*15 × 10^–3^ 1.0319 5 × 10^–1^
After merging	CO $w_{x}$ $w_{y}$	1326,813 1326,813 1326,813	3*.*48 × 10^–2^ −5*.*79 × 10^–2^ −5*.*76 × 10^–2^	7*.*15 × 10^–3^ 8*.*75 × 10^–1^ 4*.*64 × 10^–1^
After wind speed averaging, rescaling, and low-pass filtering	CO $w_{x}$ $w_{y}$	1326,813 1326,813 1326,813	3*.*48 −5*.*79 × 10^–2^ −5*.*76 × 10^–2^	7*.*15 × 10^–1^ 1*.*88 × 10^–1^ 1*.*33 × 10^–1^

Fig. 4 illustrates two examples of processed CO concentration series at representative spatial coordinates in the Jakarta Metropolitan Area. The filtering residuals approximately followed Gaussian distributions based on the Kolmogorov-Smirnov assessment.

**Fig. 4:**
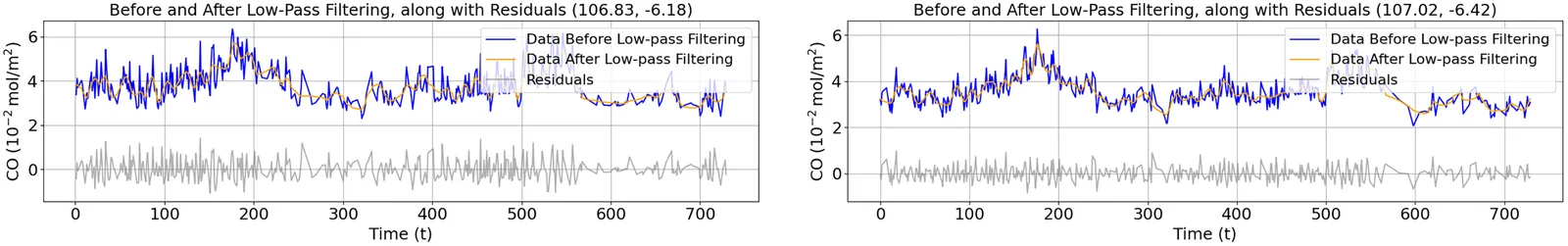
A comparison of CO levels in the Jakarta Metropolitan Area before and after low-pass filtering.

For the inverse problem, training was carried out for an average of 17.15 epochs (3889 iterations), 15.73 epochs (3644 iterations), and 16.83 epochs (3816 iterations) for the tanh, Swish, and Swish hidden layers with Softplus output configurations, respectively. The collocation and PDE losses for each model are presented in Table 3.

**Table 3:** Results of the inverse problem - a comparison of activation functions and distribution fitting for predicting the CO effective diffusion parameter in the Jakarta Metropolitan Area.

Activation Function	Collocation Points Loss	PDE Loss	Fitted Distribution (*p*-value)	Distribution Parameters	E [λ]
*tanh*	0*.*0267	2*.*1935 × 10^–7^	Burr (*p* = 0*.*8976)	*c* = 1*.*2089 × 10^5^, *d* = 0*.*1444, *loc* = −107*.*1246, *scale* = 107*.*5156	0*.*3314
Swish	0*.*0290	1*.*9800 × 10^–7^	Beta (*p* = 0*.*9312)	*a* = 19*.*7390, *b* = 1*.*6577, *loc* = −1*.*1238, *scale* = 1*.*6169	0*.*3681
Swish (hidden layers) + Softplus (output)	0*.*0275	2*.*1372 × 10^–7^	Beta (*p* = 0*.*8879)	*a* = 46*.*3360, *b* = 1*.*2789, *loc* = −2*.*9547, *scale* = 3*.*3670	0*.*3219

According to the Kolmogorov-Smirnov test, the estimated effective diffusion parameters were fitted to specific distributions for each model. The expected values (E [λ]) of these fitted distributions were used in the subsequent forward problem, as shown in Table 3. The corresponding visualizations and five-number statistics are shown in Fig. 5 and Table 4, respectively.

**Fig. 5:**
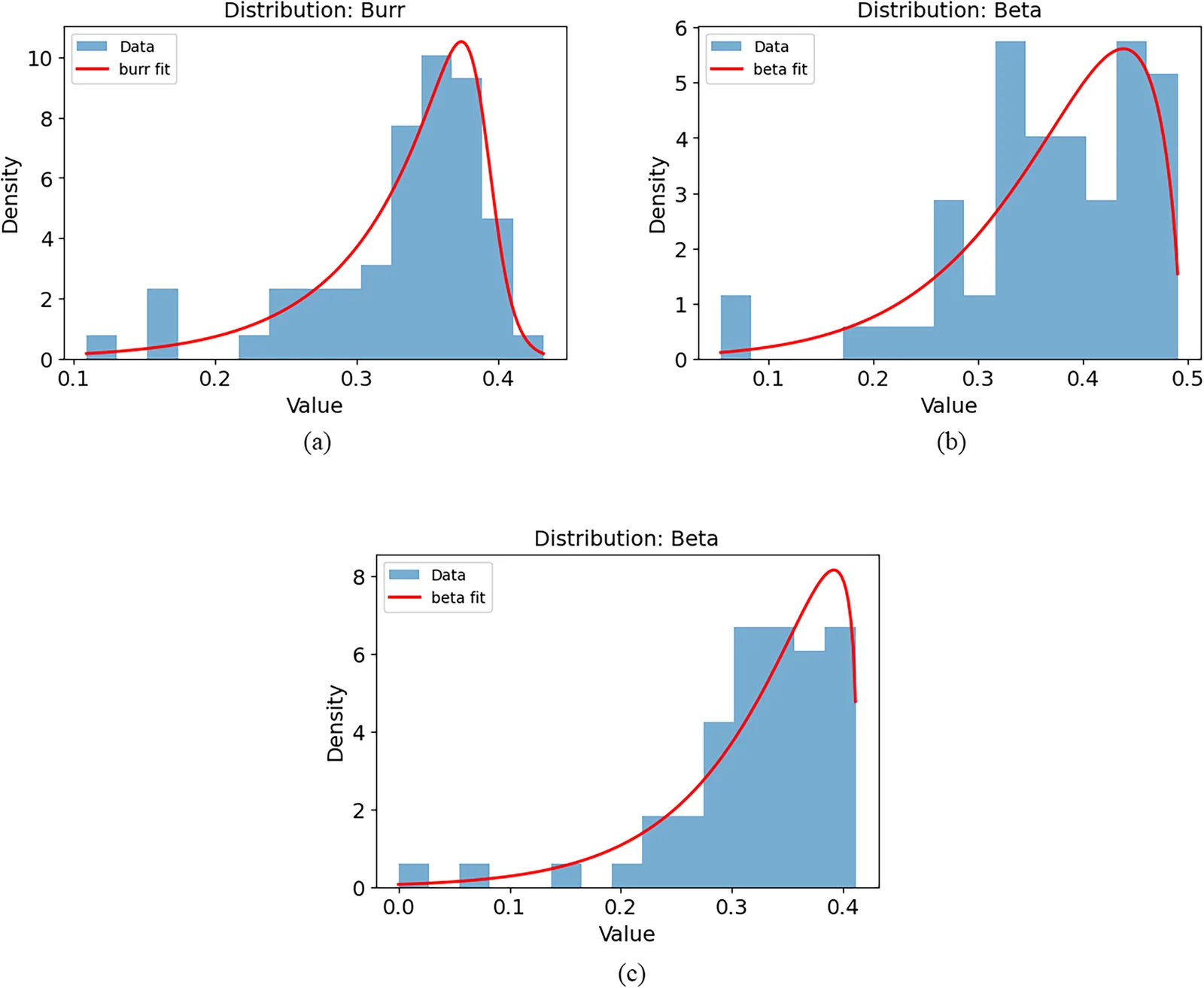
Results of the inverse problem - fitted distributions from effective diffusion parameter estimation of CO in the Jakarta Metropolitan Area using PINN architectures with different activation functions.

**Table 4:** Five-number summary of the estimated CO effective diffusion parameter λ in the Jakarta Metropolitan Area for different activation-function configurations.

Activation Function	Minimum λ	First Quartile λ	Median λ	Third Quartile λ	Maximum λ
*Tanh*	0.109203	0.318804	0.350030	0.373363	0.431640
Swish	0.054728	0.330548	0.388559	0.444904	0.489998
Swish (hidden layers) + Softplus (output)	0.000010	0.297505	0.337299	0.371941	0.411181

The estimated effective diffusion parameter varies across the activation functions. This variation occurs because λ is inferred from the derivatives of the PINN approximation rather than measured directly. Different activation functions produce slightly different fitted concentration fields and therefore different first- and second-order derivatives entering the PDE residual. As a result, the balance between fitting the observations and satisfying the PDE can lead to different estimates of λ. Nevertheless, the PDE losses are of similar magnitude (1.98 × 10^–7^ to 2.19 × 10^–7^ in Table 3), indicating that the three estimates provide comparably small PDE residuals. The variation in λ should therefore be regarded as part of the uncertainty associated with the inverse estimation.

In the forward problem, the average training loss converged to 0.1523 for tanh, 0.0831 for Swish, and 0.0693 for Swish with Softplus output. A detailed evaluation is presented in Table 5.

**Table 5:** Results of the forward problem - a comparison of MAE and MAPE for the proposed method on CO concentration prediction in the Jakarta Metropolitan Area.

Attempt	*tanh*	Swish	Swish + Softplus			
	MAE	MAPE (%)	MAE	MAPE (%)	MAE	MAPE (%)
1	0.1029	2.9786	0.0745	2.2001	0.0598	1.7561
2	0.0882	2.5530	0.0671	1.9593	0.0655	1.9197
3	0.0900	2.6472	0.0724	2.1269	0.0673	1.9760
4	0.0840	2.4609	0.0692	2.0251	0.0678	1.9953
5	0.0853	2.5095	0.0640	1.8772	0.0639	1.8668
Average	0.0901	2.6299	0.0694	2.0377	**0.0649**	**1.9028**
Standard Deviation	0.0075	0.2067	0.0042	0.1288	0.0032	0.0962
*Coefficient of Variation* (%)	8.3784	7.8592	5.9796	6.3219	4.9857	5.0558

Overall, all PINN models achieved a CV below 10%, indicating stable predictive errors across repeated runs. The Swish with Softplus output configuration delivered the best predictive performance, with an MAE of 0.0649 and a MAPE of 1.9028% on the testing dataset. Therefore, this configuration was selected for CO reconstruction and imputation.

### Reconstruction and imputation of CO

Fig. 6 presents a comparison of the observed data after preprocessing with CO concentrations obtained through two alternative outputs: imputation and reconstruction. These outputs are treated as parallel results rather than sequential steps and are evaluated during a major fire event in the Jakarta Metropolitan Area.

**Fig. 6:**
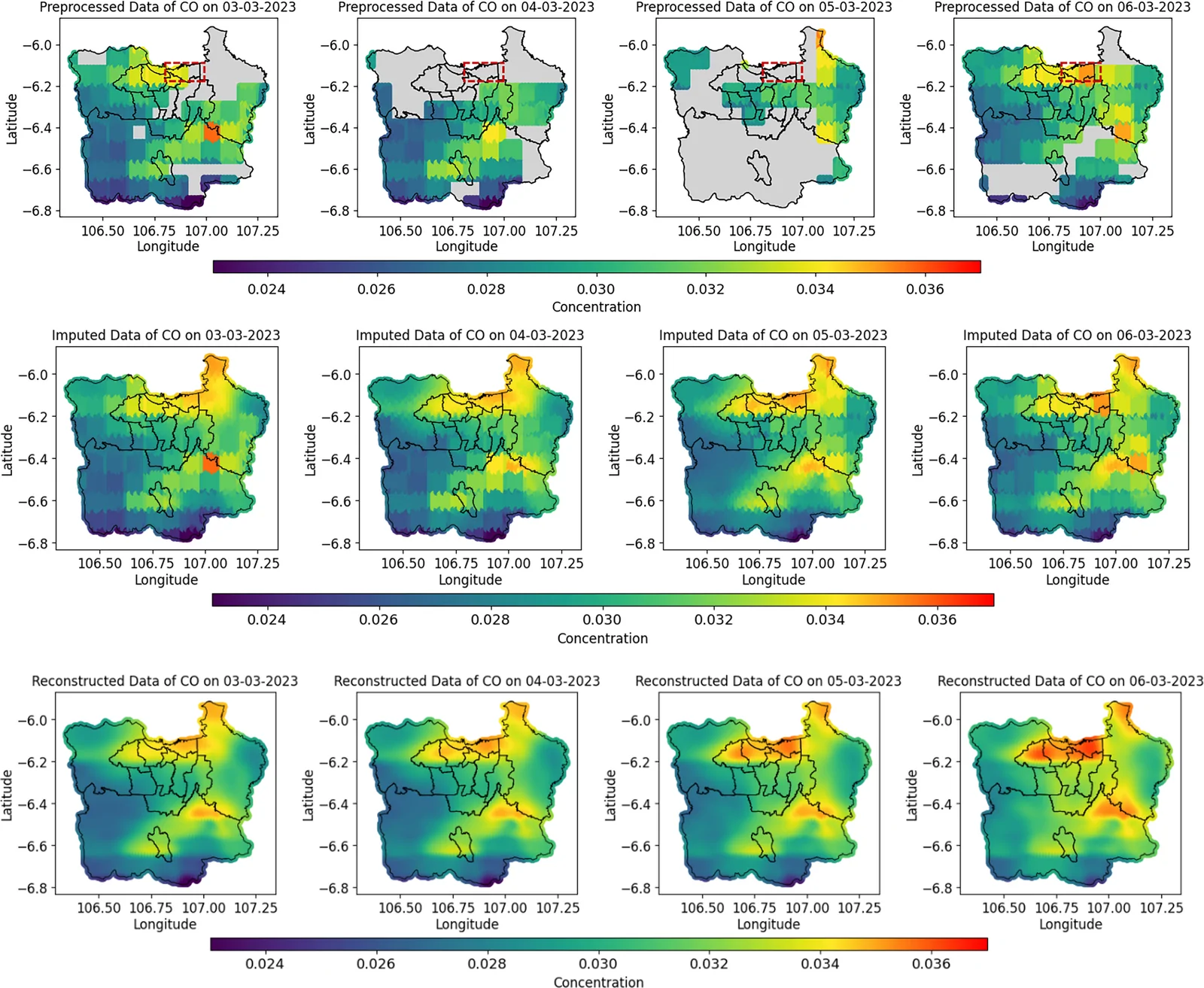
Reconstruction and imputation of CO data in the Jakarta Metropolitan Area during the 2023 Pertamina Depot fire incident using the method with Swish+Softplus activation functions.

The *Pertamina* Depot fire occurred in North Jakarta (Jakarta Utara; longitude 106.79, latitude −6.14) on the evening of 3 March 2023. The first row of Fig. 6 shows the preprocessed observations from 3–6 March. When the fire occurred within the region outlined by the crimson dotted border, the observed data did not capture a distinct increase in CO concentrations on the subsequent days (4–5 March), resulting in an incomplete representation of the pollution dynamics associated with the event.

The imputed CO fields in the second row, produced through PINN-based interpolation of missing values, show a gradual increase on 4–5 March. The reconstructed fields further suggest a localized rise in North Jakarta, reflected by the transition from yellow to red shading through 6 March 2023. The model is designed to reconstruct missing CO concentrations from the available satellite observations while accounting for transport by wind and diffusion. Although pollutant emissions can be represented by a source term in the advection-diffusion equation, such a term is not included here because the fire emission rate is unknown and is not estimated in this study. Including an unknown source term would require the model to estimate both the emission source and the effective diffusion parameter from incomplete observations, introducing an additional inverse problem beyond the scope of the proposed reconstruction framework. Therefore, the localized increase shown in Fig. 6 represents a reconstruction of the CO concentration field constrained by the available observations and the assumed transport-diffusion dynamics. Also, because no independent ground-station validation is available for this case study, the reconstructed pattern should be interpreted as a physically coherent PINN-based reconstruction rather than a directly confirmed pollution signal.

### Interpretation and evaluation

The results presented in the Method validation section show that Sentinel-5P Level-3 products may exhibit coarse spatial patterns and abrupt color transitions across the analysis domain. These features reflect the gridded nature of the satellite product and the presence of missing or uneven observations. In contrast, pollutant transport is expected to vary continuously in space and time at the scale considered in this study. Although the PINN was trained using satellite-derived observations, the reconstructed fields (the last row in Fig. 6) were smoother than the original Level-3 product. This behavior is consistent with the use of the advection-diffusion PDE constraint, which encourages physically coherent spatial-temporal variation, and with the low-pass filtering step applied during preprocessing to reduce short-term noise and volatility.

This result highlights both the limitations of the satellite-derived input data and the potential value of the PINN-based reconstruction workflow. The Jakarta Metropolitan Area case study was used to assess whether the proposed method can generate physically plausible CO fields under incomplete observations within the observed spatial-temporal domain. Because the training and testing data were obtained using a random 70/30 split, the reported validation performance represents interpolation at held-out observations within the observed spatial-temporal domain and does not evaluate extrapolation to new locations or future periods.

The multi-mode Fourier feature architecture was used to represent spatial-temporal patterns at multiple frequency scales. Among the evaluated configurations, the model using Swish activation in the hidden layers and Softplus activation in the output layer achieved the lowest MAE and MAPE.

Within this interpolation setting, the method is useful for reconstructing missing satellite observations when neighboring spatial-temporal information is available. During the 2023 *Pertamina* Depot incident in North Jakarta, the PINN produced a physically plausible CO reconstruction despite gaps in the satellite record, showing an increasing trend after the fire event. This result should be interpreted as evidence of the method’s ability to generate physics-informed reconstructions from incomplete satellite observations, rather than as independent confirmation of the true atmospheric CO fields.

## Limitations

Because ERA5-Land provides wind fields at a coarser spatial resolution than the pollution data, the wind information represents regional-scale meteorological conditions rather than fine-scale urban airflow variability. In addition, wind speed values were averaged at each spatial coordinate to maintain consistency with the advection-diffusion formulation, where wind is represented as a spatially varying but time-independent input. This simplification improves tractability but does not capture short-term meteorological variability. Because Sentinel-5P CO represents a satellite-derived atmospheric column product, the use of 10 m wind should be interpreted as a reduced-order transport proxy rather than a complete vertical transport representation.

The proposed workflow also uses a reduced-order PDE model and does not explicitly include detailed chemical reaction mechanisms, emission inventories, deposition processes, or boundary inflow-outflow conditions. Therefore, the reconstructed fields should be interpreted as physics-informed approximations of the dominant transport-diffusion behavior rather than complete atmospheric chemistry simulations.

Although the PINN formulation avoids the explicit construction of artificial boundary conditions at the edge of the analysis region, the implementation is still performed over a finite observational window determined by the available satellite and wind data. In addition, no independent ground-station CO measurements were available for direct validation of the reconstructed fire-event signal. Therefore, the event-based reconstruction should be interpreted as a physically plausible reconstruction rather than an independently confirmed pollution field.

## Ethics statements

Not relevant.

## Related research article

None.

## Specifications table

**Table:** 

**Subject area**	Mathematics and Statistics
**More specific subject area**	Data imputation and reconstruction
**Name of your method**	Air-IFF-PINN (Air-Pollution Inverse-Forward Fourier Physics-Informed Neural Network)
**Name and reference of original method**	Physics-informed neural network (PINN) [3]; Fourier feature-enhanced PINN for high-frequency and multifrequency PDE solutions [4]; Hadamard-Physics-Informed Neural Networks (H-PINN) for inverse problems [7]
**Resource availability**	The primary datasets were accessed through the Google Earth Engine (GEE) multi-petabyte catalog: - Carbon monoxide (CO) data: Sentinel-5P TROPOMI Offline (OFFL) CO. (https://developers.google.com/earth-engine/datasets/catalog/COPERNICUS_S5P_OFFL_L3_CO). - Wind speed data: ERA5-Land Hourly Reanalysis (https://developers.google.com/earth-engine/datasets/catalog/ECMWF_ERA5_LAND_HOURLY). Software & Development Environment: - Google Colab (https://colab.research.google.com/). *-* Python Libraries: Python (3.11.3), Pandas (2.3.0), NumPy (2.3.0), Datetime (Python Standard Library), Matplotlib (3.10.3), mpl_toolkits.mplot3d, TensorFlow (2.18.0), and Keras (3.8) The experiment results are provided in the supplementary material.

## Supplementary material (Data and code availability)

The original Sentinel-5P CO and ERA5-Land wind datasets are publicly available through Google Earth Engine. The processed datasets and supporting materials used in this study are permanently accessible at: http://u.pc.cd/mTF7. The experiment scripts are available at: https://github.com/nicholast25/AirPollutionPINN/tree/main.

## CRediT authorship contribution statement

**Helena Margaretha:** Conceptualization, Methodology, Supervision, Project administration, Writing – original draft, Writing – review & editing. **Nicholas Andrew Tanubrata:** Software, Data curation, Formal analysis, Investigation, Visualization, Validation, Writing – original draft. **Kie Van Ivanky Saputra:** Methodology, Formal analysis, Validation, Writing – review & editing. **Ferry Vicenttius Ferdinand:** Software, Data curation, Investigation, Visualization, Writing – original draft. **Ardhasena Sopaheluwakan:** Resources, Data curation, Validation, Writing – review & editing.

## Declaration of competing interest

The authors declare that they have no known competing financial interests or personal relationships that could have appeared to influence the work reported in this paper.

## Data Availability

I have shared the link the Retrieved Datasets in the Supplementary Materials Section. Links to data sources for other regions or pollutants are mentioned in the papers.

## Footnotes

2 https://www.sco.wisc.edu/2022/01/21/how-big-is-a-degree/
